# Prolonged Intestinal Mucosal Barium Coating due to Ischemic Necrosis

**DOI:** 10.1155/2011/193891

**Published:** 2011-09-19

**Authors:** Vincent H. S. Low

**Affiliations:** Radiology Department, InSight Clinical Imaging, 3/7 Wise Street, Joondalup, WA 6027, Australia

## Abstract

A case of a 63-year-old man with small bowel ischemia six weeks after transplantation surgery is presented. Plain abdominal radiograph obtained several days after ingestion of barium shows the sign of prolonged barium coating indicating severe mucosal damage. Abdominal CT scan demonstrates small bowel wall thickening as well as pockets of peritoneal fluid collections. Most critically, CT allows visualization of subtle traces of dense barium within the dependent portions of this fluid indicating bowel perforation.

## 1. Introduction

Recipients of transplants are a unique group of patients that pose a diagnostic dilemma when their recovery is complicated. Graft-versus-host disease (GVHD) is a potential life-threatening complication usually seen with bone marrow transplantation but has been seen with other organ transplants where immunocompetent cells are introduced into the immunosuppresed host [1, 2]. Prolonged small bowel mucosal coating with barium was first recognized in GVHD [3]. This case illustrates the sign of prolonged barium coating and suggests that this sign is evidence for severe mucosal damage in this particular case due to ischemia.

## 2. Case Report

A 63-year-old man presented six weeks after right orthotopic lung transplantation with abdominal pain suggesting an intra-abdominal catastrophe. 

**Figure 1:**
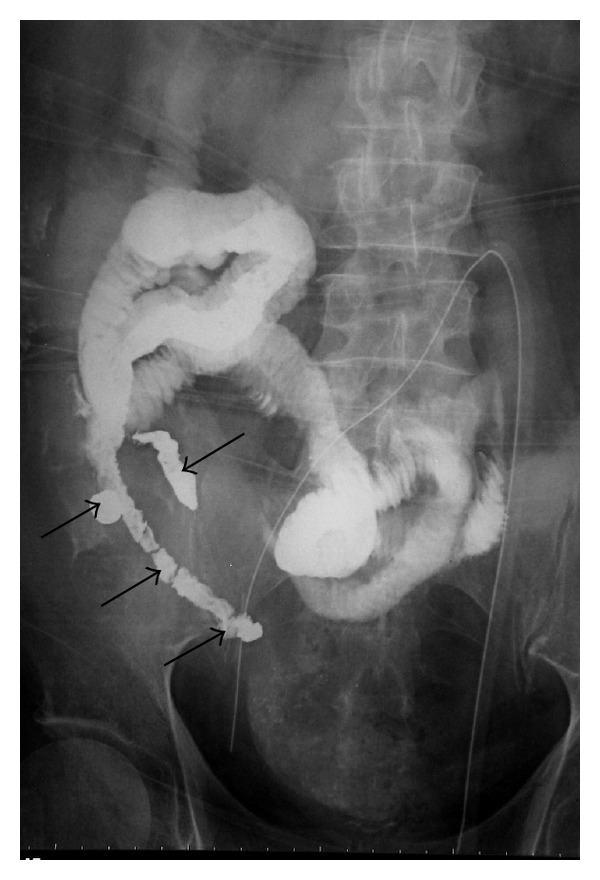
Supine abdominal radiograph. There is minimal visible bowel gas. Several loops of the small bowel are visible due to the presence of residual barium from a swallow study three days before. Segments in the right lower quadrant are particularly abnormal with the barium forming a dense cast-like appearance (arrows).

Plain abdominal radiograph (Figure 1) demonstrates a large amount of fluid within nondistended small bowel which was otherwise relatively gasless. There was curious dense opacification of the distal ileum with barium. This contrast had been administered orally three days earlier during a speech pathology swallowing evaluation of recent onset oropharyngeal dysphagia. Despite the persistence of focal small bowel opacification, the bowel was not distended to suggest obstruction or ileus as the etiology for delayed passage of contrast.

**Figure 2:**
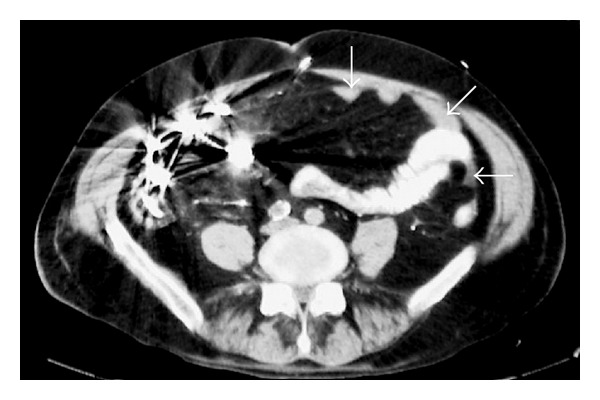
Abdominal CT scan at the level of the iliac crests demonstrates dense barium opacification of the abnormal right lower quadrant segments of ileum. The loop of small bowel leading into these segments shows increasing wall thickening. Scattered pockets of fluid (arrows) are seen elsewhere.

**Figure 3:**
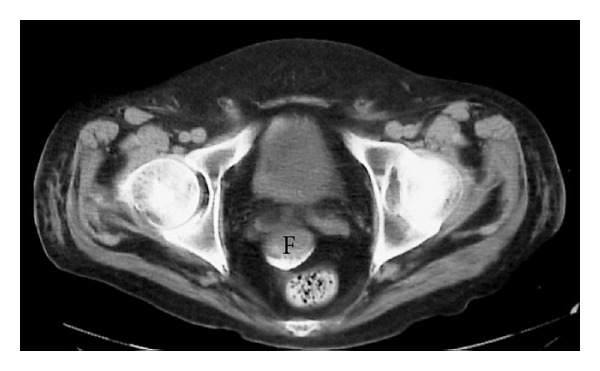
CT scan through the pelvis at the level of the acetabula demonstrates fluid (F) between the seminal vesicles and the rectum. Dense barium is present dependently within the fluid.

A subsequent abdominal CT scan (Figure 2) again demonstrates the residual barium in the distal ileum which imposes considerable streak artifact. There is associated small bowel wall thickening of this segment as well as tiny interloop extraluminal collections of fluid. Images through the pelvis (Figure 3) reveal a larger fluid collection which contained a small amount of dense barium in a dependent position indicating perforation of bowel.

With the evidence for bowel perforation, he went emergently for exploratory laparotomy. Intraoperatively, his peritoneal cavity was grossly soiled with succus entericus as well as fresh CT contrast. Examination of the bowel revealed two discrete perforations in the distal two feet of ileum. In addition, the remainder of the small bowel, cecum, and ascending colon showed blotchy and dusky areas which blanched with bowel wall thickening. Blotchy hematomas were identified throughout the mesentery. Grossly, these findings suggested ischemia which was confirmed on histologic examination with mucosal necrosis. The diseased small and large bowel were resected, and a right lower quadrant ileostomy and left upper quadrant mucus fistula were created.

## 3. Discussion

Transplant recipients are a unique subset of patients that pose a diagnostic dilemma for the referring clinician as well as the radiologist. As in this patient, the constellation of symptoms including oropharyngeal dysphagia, renal failure, and abdominal pain six weeks after transplant surgery combined with radiographic findings of initially fluid filled nondilated small bowel but persistent barium coating (in the terminal ileum) and subsequent evidence of perforation by abdominal CT scan posed the diagnosis of GVHD or possibly opportunistic infection. The time interval since transplant (six weeks) was compatible with GVHD.

GVHD is usually seen with bone marrow transplantation which may be used in the treatment of a variety of hematological disorders and malignancies. GVHD has also been seen with other organ transplants such as liver, small bowel, or heart transplant and even with blood transfusion [1, 2, 4]. Infection, especially opportunistic and viral, was a consideration as well, but all cultures subsequently returned negative and there were no rises in any viral antibody titres tested for. Ischemia was not clinically suspected. The evidence of extravasated barium on CT forced management towards exploratory laparotomy allowing the diagnosis of ischemia to be established. This paper illustrates that the sign of prolonged barium coating is evidence for severe mucosal damage in this particular case due to ischemia.

GVHD is a systemic disease when immunocompetent lymphoid tissue in the foreign donor graft mounts a reaction against the host and may involve a variety of organs most commonly the skin, liver, and gastrointestinal tract [4–6]. Graft-versus-host-disease may ultimately affect 50–70% of survivors who have undergone transplantation [3–5]. The acute form of GVHD develops 3–5 weeks after transplantation. Intestinal tract involvement has been recognized to affect any segment of the bowel from the mouth to the rectum [5, 7]. The histologic changes consist of necrosis of crypt epithelium leading to glandular depopulation [6, 8].

Patients with GVHD may complain of difficulty swallowing due to pharyngeal or esophageal pathology. Oral or pharyngeal abnormalities may occur due to functional problems, such as poor bolus control, abnormal pharyngeal retention, or abnormal mucous production or due to structural lesions, such as scarring and strictures. Esophageal abnormalities may also result in difficulty swallowing either in the form of structural lesions, such as strictures, webs, and mucosal inflammation (esophagitis), or functional abnormalities, such as weak peristalsis or incoordinate contractions (nonspecific esophageal dysmotility) [7]. 

Intestinal involvement manifests as diffuse inflammation followed by ulceration, which may be quite extensive and eventually resulting in submucosal fibrosis and stricture formation [7]. These patients usually present with secretory watery diarrhea [3–6]. Specific diagnosis may be sought by liver, rectal, or small bowel biopsy [5, 6]. These procedures however are not without significant hazard, especially in a patient with low platelet or leukocyte counts. Furthermore, changes in the biopsy may not necessarily reflect changes elsewhere in the intestinal tract. Radiological signs are therefore important in the diagnosis of GVHD.

Radiological features of GVHD include bowel lumen narrowing, mucosal fold thickening and nodularity or effacement, and bowel wall thickening, a constellation producing a characteristic featureless or “ribbon” bowel [3, 8–10]. Luminal fluid may be increased and motility altered, either rapid or delayed transit [5, 6, 8]. Changes tend to be most marked distally [6]. The main difference between acute and subacute changes is a diffuse involvement and segmental distribution later [5, 8, 10]. 

It may be difficult to differentiate clinically or radiologically GVHD involvement from other pathology, which may involve the intestines related to the patient therapy [3]. Infection is usually opportunistic, usually due to an enterovirus and occurs early in the course of the patient's disease and immunosuppression. Differentiation of infection from GVHD is important because the therapy for GVHD further depresses the patient's immunity which would worsen any infection. Radiological findings are similar in these two entities although gastric involvement is more suggestive of viral infection [3]. Prolonged small bowel mucosal coating with barium was first recognized in GVHD but has been since also been described in viral infection. This prolonged coating may also be recognized on CT scan as barium collecting in the bowel wall appearing circular in cross section or parallel tracks in longitudinal section. This is associated with severe mucosal disease and sloughing of membranes [3]. It is speculated that the prolonged coating may be due to barium adherent to mucosa, to submucosa, or to pseudomembranes, or trapped in sloughed mucosa [3].

## 4. Summary

This case highlights the value of interpreting imaging studies with the knowledge of prior studies. The recognition of the three-day time interval between the ingestion of the barium and the abdominal radiograph was crucial to the observation of prolonged barium coating. The case also discusses complications commonly considered in the post-operative course of transplant patients, including rejection, opportunistic infection, and GVHD. Furthermore, it emphasises that this patient population is at risk for additional complications that may not be directly attributable to the aforementioned problems. The etiology for this patient's bowel ischemia is uncertain. The radiological findings in this case suggest that observation of prolonged barium mucosal coating heralds severe mucosal damage. This in turn indicates a need for a close monitoring of the patient as it progresses to complete bowel wall perforation.
